# pH-Triggered Adhesiveness and Cohesiveness of Chondroitin Sulfate-Catechol Biopolymer for Biomedical Applications

**DOI:** 10.3389/fbioe.2020.00712

**Published:** 2020-06-29

**Authors:** Annachiara Scalzone, Maria A. Bonifacio, Stefania Cometa, Fabio Cucinotta, Elvira De Giglio, Ana M. Ferreira, Piergiorgio Gentile

**Affiliations:** ^1^School of Engineering, Newcastle University, Newcastle upon Tyne, United Kingdom; ^2^Department of Chemistry, University of Bari Aldo Moro, Bari, Italy; ^3^Jaber Innovation s.r.l., Rome, Italy; ^4^School of Natural and Environmental Sciences, Newcastle University, Newcastle upon Tyne, United Kingdom

**Keywords:** chondroitin sulfate, catechol, soft tissue engineering, human mesenchymal stem cells, hydrogel

## Abstract

Nature provides biomaterials that tend to be effective to control both their adhesive and cohesive properties. A catecholamine motif found in the marine mussels, the *mytilus edulis* foot protein, can play adhesiveness and cohesiveness. Particularly, acidic pH drives catechol (Cat) to have adhesive function, resulting in surface coating, while basic pH allows to enhance its cohesive properties, resulting in the formation of hydrogels. In this work, we demonstrated the usefulness of Cat-conjugated chondroitin sulfate (CS) as a platform for mesenchymal stem cell culture, utilizing the adhesive property of CS-Cat as coating for different substrates and the cohesive properties as hydrogel for cells encapsulation. To prepare the CS-Cat biopolymer, dopamine (DP) was coupled to the CS by carbodiimide coupling reaction and the Cat content was determined by UV–Vis spectroscopy (4.8 ± 0.6%). To demonstrate the adhesive properties of the biopolymer, PLA, PCL, TiO_2_, and SiO_2_ substrates were immersed in CS-Cat solution (pH < 2). Following the coating, the surfaces became highly hydrophilic, exhibiting a contact angle less than 35°. Also, in the presence of an oxidizing agent at pH 8, CS-Cat solution immediately became a hydrogel, as shown by inverted-vial test. Finally, immortalized TERT human mesenchymal stem cells (Y201) confirmed the high cytocompatibility of the biopolymer. The CS-Cat coating significantly enabled the Y201 adhesion onto PLA substrates, while the prepared hydrogel demonstrated to be a suitable environment for the encapsulation of cells as suitable bioink for further bioprinting applications.

## Introduction

Adhesive and cohesive properties are crucial features for the synthesis of functionalised materials, particularly in the biomedical field for different application, i.e., tissue engineering, cell therapy, microfluidics, etc. Adhesion and cohesion are terms often confused, although their meaning differences are deeply investigated in several books in the medical field. Adhesive means any attraction between different molecular species, brought into direct contact as the adhesive could stick or binds to the applied surface; while cohesive means attraction that occurs between similar molecular species, as results of chemical bonds between the single components of the adhesive agents (Von Fraunhofer, 2012).

Recently, biomimetic strategies based on the surface immobilization of biopolymers have been used to enhance the adhesive property to control the cellular behavior with biointerfaces created by binding biologically inert and/or active molecules on solid substrates (Neto et al., 2014; Brennan et al., 2016), improve the stability and release of biomolecules by their surface immobilization chemically or physically (Kim et al., 2015; Xu et al., 2015), or actively modify. Instead, cohesive properties have been crucial for the manufacturing of 3D scaffolds, such as hydrogels for soft tissue regeneration.

*Nature* provides biomaterials that tend to have an effective approach to control both their adhesive and cohesive properties. Considering the before mentioned requirements, various hydrogels-based bioadhesive systems have been exploited, with several inspired by marine mussels because of the adhesive strength of mussel foot proteins within a wet environment and the adaptable chemistry of catechol (Cat) groups. In particular, there is a Cat amine motif found in the marine mussels, the *mytilus edulis* foot protein (3,4-dihydroxy-L-phenylalanine and lysine), able to play adhesiveness and cohesiveness, based on the pH (Waite and Tanzer, 1981; Lee et al., 2007). Particularly, acidic pH drives Cat to have an adhesive function, obtaining a surface coating, while basic pH allows to increase its cohesive properties, resulting in the formation of hydrogels (Ryu et al., 2015). As far as the adhesiveness is concerned, Cat moieties establish strong interactions with several different mechanisms, i.e., covalent bonds, hydrogen bonds, π–π stacks, and coordination bonds (Saiz-Poseu et al., 2019; Xie et al., 2020). Conversely, in an alkaline environment, the formation of transient quinone groups leads to crosslinking reactions (e.g., dismutation and Cat–Cat bonds, hydrogen bonds, and van der Waals and other cohesive forces established with the polymeric chains), as previously reported (Yang J. et al., 2014).

The Cat moiety has been chemically included into a variety of polymers, including poly(ethylene glycols) (PEG–Cat) (Lee et al., 2010), alginate (Alg–Cat) (Lee et al., 2013), heparin (Hep–Cat) (Yang Y. et al., 2014), poly(vinyl alcohol) (PVA–Cat) (Son et al., 2013), poly(acrylic acid) (PAA–Cat) (Krogsgaard et al., 2013), dextran (Dex–Cat) (Park J. Y. et al., 2013), poly(*N*-isopropyl acrylamide) (PNIPAM–Cat) (Marcelo et al., 2014), poly(styrene) (PS–Cat) (Matos-Pérez et al., 2012), and finally chitosan (Chi–Cat) (Ryu et al., 2011; Kim et al., 2013; Han et al., 2018). Furthermore, biopolymers chemically conjugated to Cat can exhibit both adhesive and cohesive properties, based on the conditions of the surrounding environment. Hong et al. (2013) synthesized successfully hyaluronic acid (HA)-Cat conjugates and demonstrated their usefulness in neural stem cell engineering, showing an improved cells viability of the conjugated films and hydrogels compared with the systems based on HA only. Interestingly, the human neural stem cells underwent a spontaneous differentiation without mitogenic factors independently of the molecular weight of the conjugated HA biopolymers (ranging from 50 to 700 kDa). In a similar work, HA-Cat adhesive coating enhanced endothelial cell adhesion in a microfluidic-based shear assay, characterized by both hydrophilic and hydrophobic surfaces (Joo et al., 2016). Recently, properties of HA-Cat as tissue-adhesive hydrogel for enhancing biocompatibility and mediating minimally invasive cell therapy were investigated, obtaining improved therapeutic potency in pathological or defected animal models (Shin et al., 2015).

In this work, a novel chondroitin sulfate (CS)-Cat biomaterial has been engineered with bio-adhesiveness and cohesiveness, at different conditions, for regenerative medicine applications. Among the other natural polymers, CS is a less expensive proteinous natural polymer, composed of sulfated glycosaminoglycan (GAG) that is found in connective tissues, synovial fluid, hyaline cartilage, and bones, where it aids resistance toward compression. It has been described to be effective in pain relief and improvement of soft tissue regeneration, such as neural, musculoskeletal, skin (Lingor et al., 2007; Varghese et al., 2008; Salbach et al., 2012; Montell et al., 2017; Farrugia et al., 2018). Furthermore, due to its high versatility, CS has also been studied in wound healing, promoting effect and tissue regeneration after surgical operations. Recently, dopamine (DP), having a structure similar to marine mussel-secreted adhesion proteins, was found to possess strong adhesion to numerous substrates enhancing cells attachment and proliferation. The introduction of Cat groups helped to greatly enhance the adhesive ability and cytocompatibility of CS. The developed CS-Cat formulation can represent a promising candidate as bioadhesive formulation for future biomedical applications, possessing strong adhesive properties at acid pH, supporting mesenchymal stem cells viability, proliferation, and metabolic activity, via reactions with cells membrane reactive groups (amino, carboxyl, and Cat). These properties were examined through viability and cells morphology analysis. While the CS-Cat formulation at basic pH showed an excellent cytocompatibility, hosting TERT-human mesenchymal stem cells encapsulated within the hydrogel as confirmed by our *in vitro* viability tests. These results, together with the mechanical and water uptake (WU) analysis, demonstrated the potential of this hydrogel to encapsulate high cell density to be used as bioink for bioprining constructs for tissue engineering applications.

## Materials and Methods

### Materials

Chondroitin 4-sulfate sodium salt from bovine trachea (CS; MW = 515.376 g/mol), DP hydrochloride (MW = 189.64 g/mol), sodium periodate (NaIO_4_; MW = 231.89 g/mol), 2-(N-Morpholino)ethane sulfonic acid (MES; MW = 195.24 g/mol), sodium chloride (NaCl; MW = 58.44 g/mol)1-ethyl-3-(3-dimethylaminopropyl)-carbodiimide hydrochloride (EDC; MW = 191.70 g/mol), dialysis tubing cellulose membrane (MWCO = 14,000 g/mol), sodium hydroxide (NAOH; MW = 40 g/mol), N-hydroxy succinimide (NHS; MW = 115.09 g/mol), Dulbecco’s Modified Eagle Medium (DMEM), phosphate buffer saline (PBS), and ethyl alcohol solution (ETOH) were supplied by Sigma–Aldrich (United Kingdom). All the experiments were performed with the ultrapure water obtained with a Milli-Q^®^ (United Kingdom) Integral system, equipped with a BioPak^®^ ultrafiltration cartridge (Millipore, Merck, United States).

### Methods for the CS-Cat Samples Preparation

#### Synthesis of Conjugated CS-Cat

Chondroitin sulfate (5 g) was dissolved in 50 mL of an activation buffer at pH 6.0, consisting in MES 0.1 M and NaCl 0.5 M. EDC (1.82 g) and NHS (1.1 g) were slowly added to the buffer, until a final molar ratio of 1:1:1 CS/EDC/NHS was reached. DP (1.8 g) was added after 30 min of stirring, maintaining the pH constant (5.5–6) for 4 h. Subsequently, the unreacted molecules were removed by dialysis, performed for 1 day in 0.1 M MES aqueous solution, then for 2 days in acidified distilled water (pH < 2). Finally, the purified product was freeze-dried, resulting in a whitish powder. During all the synthesis steps, the solution was protected from the visible light by covering the beaker with aluminum foil. UV–visible spectroscopy (UV-1800 UV–Vis Spectrophotometer, Shimadzu) was exploited to check the Cat content in CS-Cat through its absorbance at 280 nm. In this respect, a calibration curve was built with DP standard solutions.

#### Surface Coating on Different Substrates

Chondroitin sulfate-Cat was dissolved in 1x PBS solution at a 2% (w/v) concentration; NaIO_4_ was used as an oxidizing agent, at a molar ratio of 1.5:1 with Cat. Then, the pH was reduced to 2 and 100 μL of polymer solution were deposited onto the center of the selected substrates, then spin-coated (Bench Top Spin Coater, MTI tech, Ltd.) at 1000 r/min for 3 s followed by 10 s at 7000 r/min for obtaining a homogenous thin film. This procedure was repeated twice. The selected substrates were based on glass (microscope coverslip), polycaprolactone (PCL), polylactic acid (PLA), and TiO_2_.

#### Formation of CS-Cat Hydrogels

Chondroitin sulfate-Cat hydrogels were formed by the crosslinking of Cat groups. Two different concentrations were exploited [10 and 20% (w/v)]. First, CS-Cat was dissolved in PBS at both concentrations. The pH of the solution was adjusted to 8–9 by adding 1 M NaOH. When NaIO_4_ was added, the sol/gel transition and the obtainment of a CS-Cat hydrogel happened spontaneously without any further treatment. The gelation time of the hydrogel was measured while varying the amount of NaIO_4_ (from 1.0 to 2.0 equivalents to the molar amount of Cat). The gelation time was determined when the solution flow stopped inverting the vial.

### Characterization Tests

#### Chemical Characterization by NMR, XPS, and ATR-FTIR

^1^H and ^13^C nuclear magnetic resonance (NMR) spectroscopy spectra were recorded with either Bruker AVANCE 300 MHz or JEOL 400 MHz spectrometers operating at 25°C. Samples were prepared in 5 mm NMR tubes by dissolving the compounds in appropriate deuterated solvents. Chemical shifts are reported in ppm relatively to TMS as internal standard.

X-ray photoelectron spectroscopy (XPS) was carried out to study the elemental composition of the CS-Cat conjugate. A PHI 5000 VersaProbe II (Physical Electronics, United States) was exploited, with an AlKα X-ray radiation source. The freeze-dried samples were examined recording survey scans (0–1200 eV) and high-resolution signals in Fixed Analyzer Transmission mode (pass energy 29.35 eV), scanning areas of ∼1400 × 200 μm. The MultiPak software (v. 9.9.0) was exploited for data mining. The surface elemental composition was assessed after normalizing each peak area, referring to the software library for elemental sensitivity factors. High-resolution spectra were fitted with Gaussian–Lorentzian peaks having the same FWHM.

Attenuated total reflection-Fourier transform infra-red (ATR-FTIR) analysis was performed on a Spectrum Two PE instrument using the Universal ATR accessory (Single Reflection Diamond) (PerkinElmer Inc., Waltham, MA, United States) in a range of 4000–550 cm^–1^ (resolution 4 cm^–1^). Dried samples were analyzed without any preliminary preparative step.

#### Contact Angle Measurements

Static contact angle analyses were performed with a CAM 200 KSV instrument (KSV Instruments, Finland), using the software Drop Shape Analysis System DSA 10 (V2.0-02, KRUSS GmbH, Germany). Distilled water drops (3 μL) were used for each analysis, repeated at least three times per sample. Results were expressed as mean ± standard deviation.

#### Morphological Analysis by Scanning Electron Microscopy

Hydrogels morphological analysis was performed on freeze-dried samples [lyophilization took place for 48 h (Christ ALPHA 1-2/LD Plus, Martin Christ, Germany)] by scanning electron microscopy (SEM, HITACHI TM3030, Maidenhead, United Kingdom). The diameters of the pores were evaluated on five SEM micrographs using ImageJ software.

#### Water Uptake

The WU test was performed on the freeze-dried samples. Each sample was weighted and placed separately in a 5 mL vial with the addition of 3 mL of PBS and stored at 37°C. The weight of all the samples was measured before the immersion (W_i_) and at different time points (W_t_): 30 min, 1, 2, 4, 6, 24, and 48 h of incubation. At each time point, the samples were weighted, after gently drying the extra PBS on the surface using tissue papers. The WU percentage was calculated using the following equation at each time point:

(1) $$WU\left(\%\right)=\frac{W_{t}-W_{i}}{W_{i}}\times 100$$

#### Mechanical Properties by Unconfined Compression Test

Unconfined compression tests were performed in triplicate on 10% w/v and 20% w/v CS-Cat samples using a universal testing machine (EZ-SX, Shimadzu, Japan) provided of a 20 N load cell and crosshead speed set at 1 mm⋅min^–1^. The measurement was stopped once the strain reached the 35%. Then, the stress/strain (σ/ε) curves, for both sample types, were plotted and the respective compressive Young’s moduli (E) were calculated as the slope of the initial elastic region of the curves (0–10% strain).

### *In vitro* Biological Tests

#### Cell Culture

The immortalized cell line Y201 derived from human mesenchymal stem cells (Y201) were kindly provided by Prof. P. Genever (York University) and cultured as already described (James et al., 2015). Cells were grown at 37°C, 5% CO_2_, in DMEM with low glucose content, with the addition of 10% fetal bovine serum (FBS), 2 mM L-glutamine, and a 1% penicillin–streptomycin (P/S) mixture (100 U/mL).

#### Cell Viability and Morphological Analysis Assessment on the Coated Substrates

The biological behavior of Y201 cells was examined on PLA-CS-Cat spin-coated substrate and compared with uncoated PLA substrate and tissue culture plate (TCP). Prior to cells seeding, all samples were sterilized with 70% ETOH for 20 min and treated with Sudan Black (SB) to limit auto fluorescence. Each sample was covered with 50 μL of SB solution [0.3% (w/v) in EthOH], incubated for 20 min at 37°C and washed properly twice with PBS. Then samples were sterilized under a UV lamp for 30 min and placed in 48-well plate. The cell density was 5 × 10^4^ cells per sample. Cells viability was studied via Live/Dead staining (Live/Dead Cell Staining Kit II, PromoKine, PromoCell GmbH, Germany) after 1 and 3 days of culture for each sample, following the manufacturer’s protocols. In this kit, calcein AM and ethidium bromide are combined to provide two-color discrimination based on the cells state: live cells in green and dead cells in red. All the samples were washed with PBS and the staining solution, prepared diluting 4 μM ethidium homodimer-1 and 10 μM calcein in PBS, was added to the samples. Following this, samples were incubated for 30 min at 37°C and images were collected at each time point using a fluorescence microscope (EVOS M5000).

To assess the cells metabolic activity, the culture medium was removed at each time point (1 and 3 days), samples were washed with PBS and 1 mL of PrestoBlue stock solution, obtained from a dilution (1:10) of PrestoBlue^TM^ reagent (Thermo Scientific, United Kingdom) in DMEM, was added to each well and incubated for 2.5 h at 37°C and 5% CO_2_. Then, 200 μL of each well solution (in triplicate) was transferred to a 96-well plate and a spectrophotometer (FLUOstar OMEGA microplate reader) was used to measure the fluorescence (excitation/emission of 560/590 nm). Following, samples were washed with PBS and fresh DMEM was added for the next time point. All this experiment was performed in light-covered conditions (Scalzone et al., 2019). To evaluate the cells morphology and their adhesion to the substrates, immunostaining analysis were performed. Samples were fixed in pre-warmed 4% w/v paraformaldehyde (PFA) and cells were permeabilized with 0.1% (v/v) Tween20^®^ in PBS. Rhodamine-phalloidin solution was prepared using phalloidin-tetramethylrhodamine B isothiocyanate (1:1000 in 0.1% PBS/Tween20^®^) for 30 min at room temperature. Then, samples were washed with 0.1% PBS/Tween20^®^ and DAPI solution (Vector Laboratories, United Kingdom) was added (1:2500 in 0.1% PBS/Tween20^®^) for 15 min at room temperature. Images were collected at 7 days using a fluorescence microscope (EVOS M5000).

#### Cell Encapsulation Within the CS-Cat Hydrogel

The biological behavior of Y201s was even examined within CS-Cat hydrogels. Prior to cells encapsulation, CS-Cat freeze-dried powder was sterilized under a UV lamp for 30 min. For the cell encapsulation CS-Cat 20% (w/v) solution (100 μL) was poured into a 24-well plate membrane-based cell culture insert (membrane pore size of 8.0 μm, Merck, Millipore, Germany) at room temperature. The same procedure explained before for the hydrogel preparation was followed and Y201s were added before the NaIO_4_ addition and mixed gently with the hydrogel solution at an optimized cellular density of 2 × 10^6^ cells/mL (Liu et al., 2017). Finally, 1 mL of fresh DMEM was added to each well and changed three times per week. Samples were stored in the incubator at 37°C, 5% CO_2_.

#### Biological Assessments: Metabolic Activity and Viability of Encapsulated Cells

The Live/Dead staining was exploited to study cell viability at day 1 and day 3. The experiment was performed as explained before for the coated substrate and samples were imaged with Nikon A1R inverted confocal microscope. To assess cells metabolic activity, the culture medium was removed at each time point (1, 3, and 7 days) and samples were washed with PBS. Then, 1 mL of Alamar Blue solution obtained from a dilution (1:10) of Alamar Blue^TM^ reagent (Thermo Scientific, United Kingdom) in DMEM protected from light, was added to each well with the gel and incubated for 2.5 h at 37°C and 5% CO_2_. Then, 200 μL of each well solution (in triplicate) was transferred to a clear bottom 96-well plate and a filter-based multi-mode microplate reader was used to measure the absorbance at 630 nm. Following, samples were washed with PBS twice and fresh media was added for the next time point. For the immunostaining analysis, samples were fixed as explained previously and DAPI staining was performed. Images were collected at 3 and 7 days using a Nikon A1R inverted confocal microscope.

### Statistical Analysis

The statistical significance of the obtained results was assessed using GraphPad Prism Software (v. 8.4.1). One-way ANOVA with repeated measurements was performed for each experiment. Then, Tukey’s *post hoc* test was carried out to highlight the main factors determining data variability. Statistical significance was set at ^∗^*p* < 0.05 and ^****^p < 0.0001.

## Results

### CS-Cat Synthesis

**FIGURE 1:**
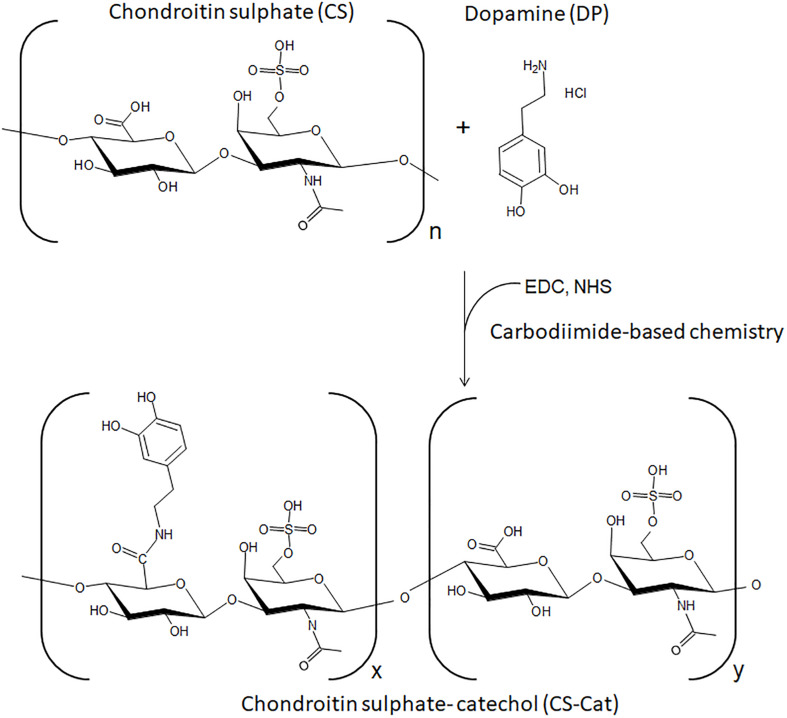
Schematic representation of CS-Cat conjugates and reaction process.

To prepare the CS-Cat conjugate, EDC coupling reaction was used to bind DP with the carboxyl group of CS as shown in Figure 1. The reaction occurred for 4 h in an aqueous activation buffer at constant pH (5.5–6).

**FIGURE 2:**
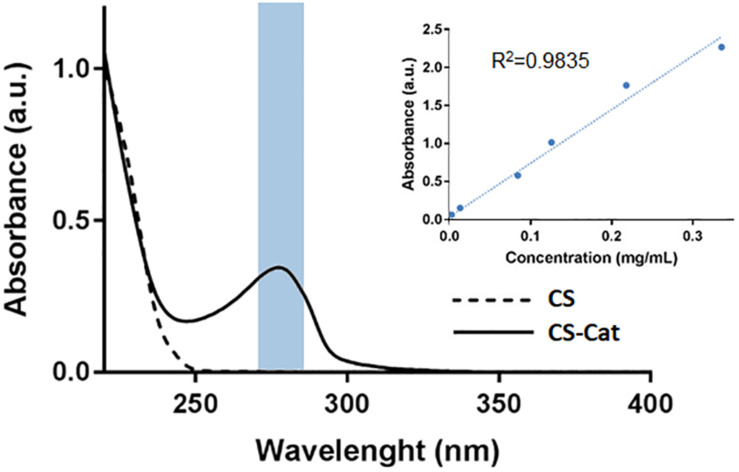
Catechol quantification by UV–Vis analysis, with a peak at 280 nm. The lack of peak after 300 nm demonstrated that the CS-Cat was not oxidized.

The results obtained by UV–Vis spectroscopy are reported in Figure 2. It can be observed at 280 nm a single absorbance peak, relative to the DP (Chen and Peng, 2003), that was not evident in the pure CS sample. The Cat content was determined at 280 nm and, comparing with the DP standard calibration curve, it resulted that approximately 4.8 ± 0.6% (*n* = 3) of the carboxylic acid groups in the CS chain were conjugated with DP. Moreover, no additional peaks at wavelengths higher than 300 nm were observed, showing that the conjugated Cat was not oxidized.

**FIGURE 3:**
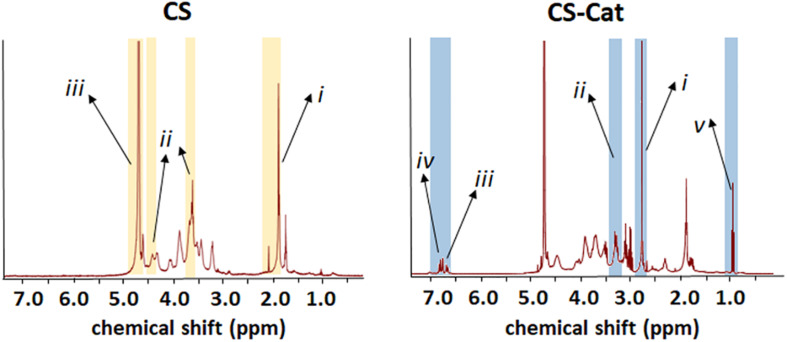
Chemical properties of synthesized CS-Cat materials: NMR spectra of CS-Cat (on the **right**) and the CS as control (on the **left**).

Different methods were performed to determine the Cat grafting and content. Indeed, the product of the synthesis of the conjugate reaction was analyzed by NMR spectroscopy and XPS. Comparing the two obtained NMR spectra (Figure 3), the CS sample presents some peaks between 1.9 and 2.3 ppm: (*i*) representative of the *N*-acetyl methyl group typical of CS, two peaks at 3.6 and 4.43 ppm, (*ii*) representative of the D-glucuronic acid and at 4.6 and 4.75 ppm, and (*iii*) representative of the *N*-acetyl-D-galactosamine, which are specifics of the chondroitin-4-sulfate (Toida et al., 1993; Üstün et al., 2011). All these peaks were re-found in the CS-Cat spectra, with the additions of new peaks: the multi-peaks centered at d 2.75 ppm (*i*) and d 3.2 ppm (*ii*) in the CS-Cat spectrum represent the two methylene groups brought by DP after they were grafted onto the CS backbones (Imperiale et al., 2013). The multi-peaks centered at 6.75 ppm (*iii*) and 6.89 ppm (*iv*) represent the corresponding methine groups located on the benzene ring of grafted DP. After the chemical reaction between CS and DP, there was still a small amount of NHS residue in the resultant CS-Cat polymers (peaks centered at d 0.8–0.9 ppm; *v*).

X-ray photoelectron spectroscopy data, reported in Table 1, compared the surface elemental compositions of pure DP, CS, and CS-Cat adduct.

**TABLE 1:** surface elemental composition of DP, CS, and CS-Cat.

Sample	Atomic %					
	
	*C1s*	*O1s*	*N1s*	*Cl2p*	*Na1s*	*S2p*
DP	68.8	16.0	8.1	7.1	–	–
CS	51.0	36.1	5.8	0.6	4.3	2.2
CS-Cat	56.7	30.3	7.8	0.9	2.6	1.7

The resulting elemental composition analysis of DP indicates that the experimentally measured C/O and C/N ratios were 4.3:1 and 9.7:1, respectively. These values are consistent with the stoichiometric ratios expected for the Cat molecule (i.e., 4:1 and 8:1, respectively), taking into account the adventitious hydrocarbon contamination onto all the surfaces. The XPS compositions of CS and CS-Cat surfaces resulted quite similar in terms of detected elements.

**FIGURE 4:**
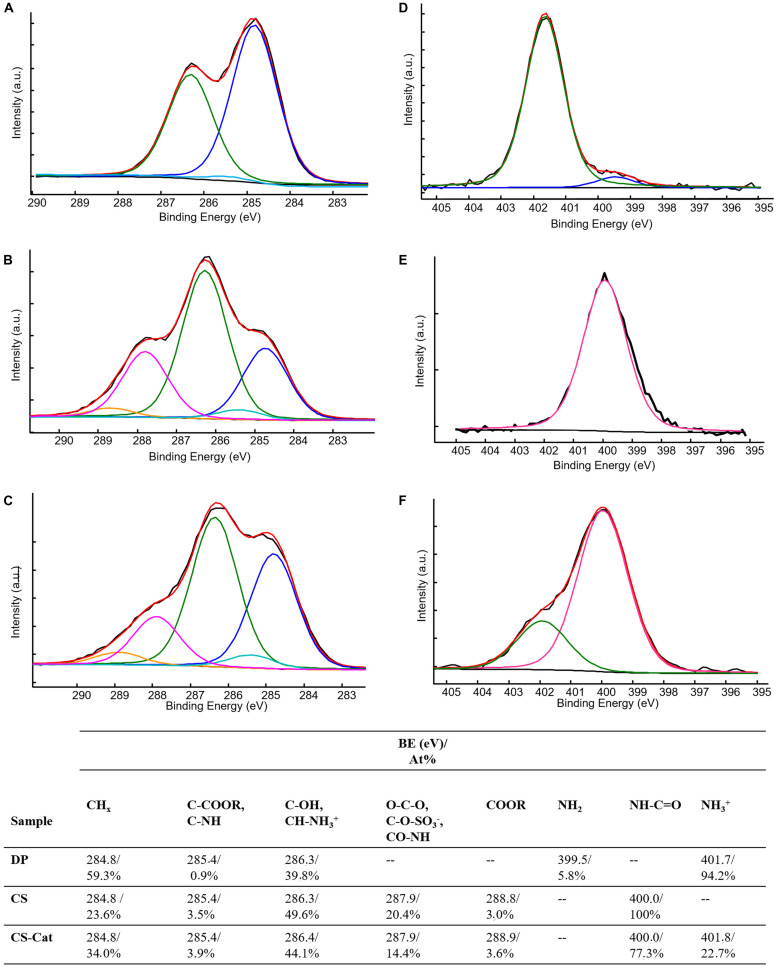
C1s **(A–C)** and N1s **(D–F)** high-resolution spectra and relevant curve fittings of DP **(A,D)**, CS **(B,E)**, and CS-Cat **(C,F)** specimens. Attributions, binding energies, and atomic percentages are reported in the table.

Typical high-resolution XPS C1s spectra for DP, CS, and CS-Cat are shown in Figure 4. The carbon peaks detected in the C1s spectra and the relevant percentages are summarized in Figures 4A–C. In DP C1s signal (Figure 4A), three contributions were detected: the first one, at 284.8 eV, typical of hydrocarbons present in the aromatic ring; a second very low peak, at 285.4 eV, relevant to C-N contribution; the third one, at 286.3 eV, ascribable both to C-OH and C-NH_3_^+^groups. Indeed, DP was present mainly in the hydrochloride form, as confirmed also by the almost similar chlorine and nitrogen atomic percentages (Table 1).

As far as the CS C1s curve fitting (Figure 4B), a typical polysaccharide carbon shape was observed, deconvoluted by five components. The first one at 284.8 eV, due to hydrocarbon contamination; the second at 285.4 eV, ascribable to carbon in α-position to carboxylic moieties; the third, typical of C-OH and C–O–C bonds present in carbohydrates; the forth at 287.9 eV, containing the anomeric carbon (O–C–O), the C–O–SO_3_^–^, and the carbon in the amide (C = O–NH) present in CS; finally, the fifth contribution, relevant to carboxylic carbon (C = O–OR).

When the CS-Cat adduct was formed, the C1s curve fitting (Figure 4C) presented similar contributions to CS sample, except for the relative atomic percentages of each contribution. Curve fitting of high-resolution N1s spectra was performed as well. Figures 4D–F show N1s curve fitting of DP, CS, and CS-Cat spectra. The relevant binding energies and atomic percentages are reported in the table. N1s spectrum of DP (Figure 4D) exhibited almost protonated amine groups, evidenced by the peak at 401.7 eV, with a low presence (about 6%) of neutral amine groups, in good agreement with C1s curve fitting. CS specimen (Figure 4E) presented only amide contribution, falling at 400.0 eV, as expected. Finally, CS-Cat (Figure 4F) presented two contribution: the first one, at 400.0 eV, due to amide groups already present in CS in addition to those created from the DP grafting; the second, falling at 401.8 eV, ascribable both to unloaded DP and to residue ester linkage between NHS and CS.

### CS-Cat as Tissue Adhesive Coating

#### Physico-Chemical Characterization

**FIGURE 5:**
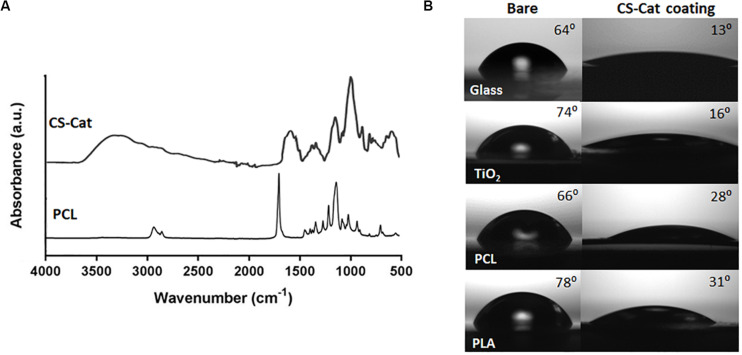
Physico-chemical modification of different substrates, i.e., glass, synthetic polymers (PCL and PLA), and metal oxides (TiO_2_) with CS-Cat coating. **(A)** FTIR-ATR spectrum of the bare PCL surface and after coating with CS-Cat. **(B)** Static contact angle analyses of the four substrates (on the left) and after CS-Cat coating (on the right).

To demonstrate the ability of the CS-Cat to coat any surface, substrates in PLA, PCL, TiO_2_, and SiO_2_ were covered by spin-coating with a CS-Cat solution at pH < 2, as reported before in Section “Surface Coating on Different Substrates,” and then washed with distilled water. The CS-Cat coating thickness was ranging from 25 to 40 μm measured by SEM analysis. The surface was characterized by infrared spectroscopy (FTIR-ATR). Figure 5A shows the spectrum of the CS-Cat coating on the PCL sample. Methylene groups (–CH_2_) were observed at 2940–2920 and 2860–2850 cm^–1^, while the amide C = O stretching signals fell at 1700–1250 cm^–1^. Carboxylate moieties (COO^–^) belonging to salts were detected at 1650–1500 cm^–1^, while the NH bending signal was recorded at 1560–1530 cm^–1^. The primary –OH-stretch occurred at 3640–3630 cm^–1^, beyond the signal at 3350–3250 cm^–1^. Ethers’ contribution (–C–O–C–) was observed at 1100 cm^–1^.

In addition, contact angle analyses were exploited to characterize surface physico-chemical changes due to CS-Cat. The latter induced a hydrophilic feature, with a considerable decrease in the contact angle of the coated substrates compared to the bare ones: SiO_2_ (from 64.2° ± 3.4° to 13.5° ± 2.7°), PCL (from 66.1° ± 3.8° to 28.2° ± 3.8°), PLA (from 78.2° ± 2.6° to 31.4° ± 2.9°), and TiO_2_ (from 73.7° ± 1.3° to 16.3° ± 1.1°) (Figure 5B).

#### Biological Assessment: Cells Viability, Metabolic Activity, and Morphology Onto the Substrates

**FIGURE 6:**
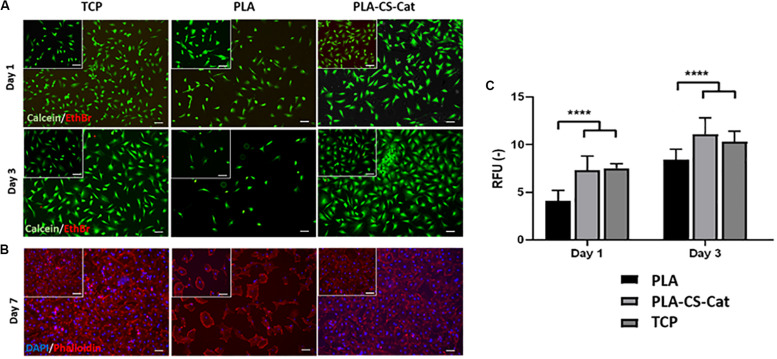
**(A)** Live/Dead images at day 1 and day 3 for the TCP, PLA, and CS-Cat coated PLA samples: Ethidium bromide (EthBr) stains dead cells (red) and calcein stains live cells (green). **(B)** Immunostaining assay images at day 7 for the TCP, PLA, and PLA-CS-Cat substrates: DAPI stains nuclei (blue) and Phalloidin stains the cytoskeleton (red). **(C)** Prestoblue test to assess cells metabolic activity at day 1 and day 3 in all the three samples (PLA, PLA-CS-Cat, and TCP). Bars: 100 μm. Statistics: ****p < 0.0001.

Live/Dead staining assay highlighted cell viability after 1 and 3 days of incubation on the CS-Cat coated PLA substrates (Figure 6A). All the samples showed a predominance of live cells (green) compared to dead cells (red) at both time points; moreover, the number of cells in the TCP and PLA-CS-Cat was higher compared to the PLA bare sample. The nuclei (DAPI) and cytoskeleton (Phalloidin) immunostaining test (Figure 6B) confirmed this statement: onto the PLA-CS-Cat samples, a higher percentage of cells was detected after 7 days concerning the PLA substrate and comparable with the TCP. Cells were assuming a spread shape on all the substrates. The PrestoBlue assay demonstrated that, from day 1 to 3, Y201s metabolic activity increased in all the samples (Figure 6C). Interestingly, cells on coated PLA substrate and TCP showed a significant higher (*p* < 0.0001) metabolic activity compared to the bare PLA substrate at each time point.

### CS-Cat as Cohesive Hydrogel

#### Physico-Chemical and Mechanical Characterization

In the presence of an oxidizing agent, sodium periodate in alkaline condition (pH 8–9), the CS-Cat solution instantly became gel by the chemical crosslinking between the conjugated Cat moieties. Particularly, we found that when the amount of sodium periodate was equivalents to the molar amount of Cat led to a complete gelation in 10 min, while when the NaIO_4_/Cat stoichiometric ratio was 2:1 we found an immediate gelation that allowed several difficulties to pour the gel in appropriate vial or wells for the following characterization. Therefore, with a molar ratio of 1.5:1 the CS-Cat (10% w/v) became a gel in 50–60 s and CS-Cat (20% w/v) in less than 30 s and both time were suitable for handling the solutions.

**FIGURE 7:**
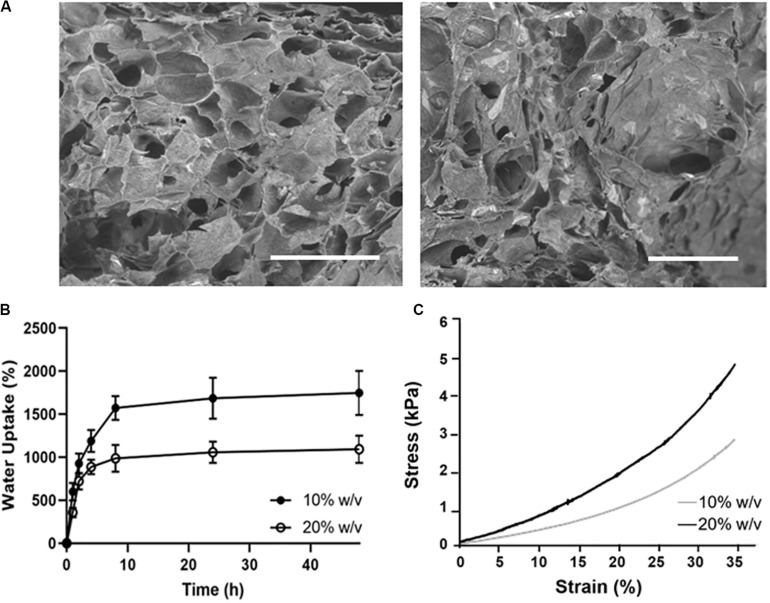
Physico-chemical characterization of CS-Cat hydrogels: **(A)** SEM images of the lyophilised CS-Cat hydrogels: on the left 10% CS-Cat and the right 20% CS-Cat. Bars = 500 μm. **(B)** Water uptake study of CS-Cat at different time points for the 10% (w/v) and 20% (w/v) concentrations. **(C)** Undefined compression test: stress/strain (σ/ε) curves for 10% (w/v) and 20% (w/v) CS-Cat hydrogels.

The porous structure and pore size of the obtained hydrogels were examined by SEM after the freeze-drying (Figure 7A). The average pore size calculated by using ImageJ software was ∼140 ± 67 μm for the 10% (w/v) CS-Cat, while the 20% (w/v) showed a non-statistically decrease (∼93 ± 58 μm).

The WU ability of the CS-Cat hydrogels is shown in Figure 7B. The samples displayed a fast increase of WU, reaching the values of 1571 ± 139% for the 10% (w/v) and 987 ± 157% for the 20% (w/v) within 10 h. Then, the WU percentage slowly increased and stabilized at 48 h at the values of 1747 ± 257% for the 10% (w/v) and 1092 ± 159% for the 20% (w/v).

Figure 7C shows the stress/strain curves for the two concentration of CS-Cat hydrogel tested. Under static compression, it was calculated the compressive elastic Young’s modulus in the linear regions of both curves (0–10% strain). The values obtained were: 4.6 ± 1.8 kPa for the 10% CS-Cat and 7.8 ± 1.0 kPa for the 20% CS-Cat.

Due to the lower WU degree and higher mechanical properties, the 20% (w/v) CS-Cat hydrogel was selected for the further tests. Furthermore, the *in vitro* degradation behavior of 10 and 20%w/v samples was also studied (Supplementary Figure S2).

#### CS-Cat as Hydrogels for Hosting Cells: Cells Viability, Morphology, and Metabolic Activity

**FIGURE 8:**
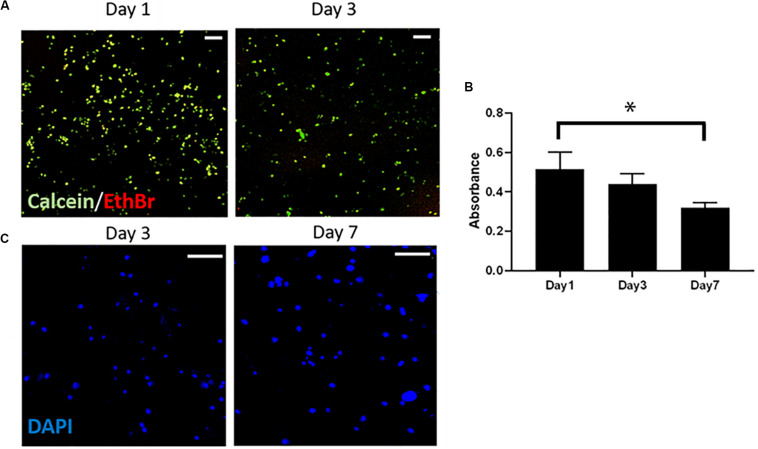
**(A)** Live/Dead test to assess the cytotoxicity of the CS-Cat hydrogel. Live cells are stained in green (calcein) and dead cells in red (ethidium bromide). Bar = 100 μm. **(B)** Alamar blue test results showing cells metabolic activity within the hydrogel upon to 7 days. **(C)** Immunostaining images taken after 3 and 7 days of culture: nuclei are stained in blue (DAPI). Bar = 200 μm. Statistics: ^∗^*p* < 0.05.

The Live/Dead staining showed that the Y201 cells encapsulated within the 20% CS-Cat hydrogels were viable (green) and characterized by round-like morphology (Figure 8A) at day 1 and day 3 of culture. Few dead (red) cells were found. Cells metabolic activity slightly decreased from day 1 to day 7 (*p* < 0.05) as shown in Figure 8B. Finally, immunostaining analysis (nuclei stained with blue DAPI) showed the cells tend to agglomerate during the culture time Figure 8C.

## Discussion

In this work, we developed a biopolymers-based formulation composed of CS, a sulfated GAG made by an alternating sugars chain (*N*-acetylgalactosamine and glucuronic acid) that generally comes from animal cartilage and DP, an organic chemical of the catecholamine and phenethylamine families, synthesized in plants and most animals (Zhu et al., 2019). Sodium periodate is an inorganic salt, composed of a sodium cation and the periodate anions and it is used for the crosslinking the conjugated CS-Cat. In particular, the Cat shows adhesive function in acidic condition, resulting in surface coating, while basic pH allows to enhance its cohesive properties, resulting in the formation of hydrogels (Saiz-Poseu et al., 2019).

The CS-Cat macromer was manufactured through a single step coupling reaction between the carboxyl groups from CS and the amine group from DP. Considering that the CS is a linear hetero-polysaccharide and holds repeating disaccharide units of glucuronic acid and galactosamine, enough carboxyl groups could be involved in the reaction. The reaction was performed in quite mild conditions without the addition of any organic or toxic reagent during the synthesis. Then, the solution obtained was dialysed against distilled water for a few days and freeze-dried; the obtained powders should be non-toxic. NMR spectra reported in Figure 3 revealed the presence of the multi-peaks related with the addition of the chemical DP groups onto the CS backbone; furthermore, a small amount of engrafted NHS residue was detected. From recent works, the NHS presence was introduced as a functional group in some bioadhesive formulations to assist the adhesion between the adhesive formulations and the biological surfaces (Strehin et al., 2010). Therefore, in the newly developed CS-Cat biomaterial, a tiny amount of NHS residue within the CS-Cat macromers can be considered acceptable.

Few conclusions relevant to the DP grafting could be obtained from the comparison of the C1s spectra, since the new bond formed between CS and DP was an amidic bond, still present on CS (Figure 4). Moreover, the possible residue presence of NHS may further complicate the curve fitting interpretation. Indeed, the amide groups in the NHS moieties were expected to fall at binding energies similar to the carboxyl groups rather than to the typical amide binding energies, probably due to the strain in the five-membered ring and the electron conjugation in the N-succinimidyl esters, as reported in the literature (Cheng et al., 2007). In this scenario, the grafting of DP to CS may improve the peak at 287.9 eV, while the residue presence of bonded NHS may enrich the peak at 288.9 eV, making impossible an undoubtedly interpretation to the fitting results. On the other hand, the fitting of nitrogen signals supplied evidence of the DP grafting on the CS backbone. Indeed, subtracting the contribution at higher BE of CS-Cat N1s curve fitting to the total nitrogen percentage, the N/S corrected peak area ratio in CS-Cat resulted equal to 3.5:1. In pure CS, this ratio was 2.6:1. The increase of this ratio can be directly ascribed to the formation of new amide groups between CS and DP, since both the free DP and the NHS contributions to the nitrogen have been excluded.

Hong et al. (2014) reported that facile surface modification of a variety of substrates in acidic condition could be obtained by Cat-functionalised biomaterial thanks to their adhesive properties. The synthesized CS-Cat exhibited an outstanding bio-adhesive property on different substrates at low pH. Figure 5 evidences the significant decrease of the contact angle of the different substrates after being coated, by spin-coating with the bioadhesive CS-Cat solution exhibiting a static contact angle < 35°. In our work, it is noticeable that most functionalized surfaces showed very hydrophilic properties and some substrates, i.e., SiO_2_ and TiO_2_ evidenced super-hydrophilic properties, where nearly 100% of the surface was covered by the immobilized CS-Cat. As reported, other materials, such as PCL and PLA, showed a contact angle close to 30°. These results might represent partial surface modification as reported also by Hong et al. (2013), where they coated different polymeric, metallic, and ceramic substrates with HA conjugated chemically with DP. Furthermore, in order to evaluate the bonding strength of the Cat-based polymers on different substrates, it is proved that *3,4-dihydroxyphenylalanine* in adhesive proteins, secreted by marine mussels, plays an important role in interfacial binding and intermolecular cross-linking (Lee et al., 2011). It was reported also that Cat could form strong and reversible bonds with metal oxides reaching 40% of covalent bond strength, the strongest reversible bond involving a biological molecule reported to date. Furthermore, as reported by Lih et al. (2016), natural polymer-Cat conjugates, when coated, start first to be self-assembled and aggregated due to relatively balanced hydrophilic (natural polymer) and hydrophobic (DP) segments, followed by π–π stacking of aromatic rings in Cat and, lastly, characterized by self-polymerisation of DP. Hovewer, the Cat amount conjugated to polymeric chain and the manufacturing method used for the coating preparation can affect the bonding strength on the substrate.

The wettability/hydrophilicity degree of the substrate surfaces strongly influence the cells adhesion and spreading with an enhanced biological behavior on hydrophilic surfaces compared to the hydrophobic ones. This is reported by several researchers (Webb et al., 1998; Dowling et al., 2011; Dave and Gomes, 2019). Among the hydrophilic surfaces, cells attachment resulted to be influenced by substrates charge and wettability, while cell area, shape, or cytoskeletal organization did not result to be affected by these properties. Surfaces with a moderate hydrophilicity (20–40° water contact angle) resulted to highly promote cells attachment. This was confirmed in our work; in fact, the Live/Dead assay showed good cells viability with few dead cells at both time points, meaning that the culture conditions did not present cytotoxic effect. Despite the Y201 showed viability even on the PLA substrate, the CS-Cat coated PLA samples showed a higher ability to promote cell attachment, spreading, proliferation and metabolic activity, compared to the PLA bare substrate (Figure 6). Indeed, Live/Dead and immunostaining assay images demonstrated that cells were spread and homogeneously distributed along all the CS-Cat coated PLA substrate surface, as well as on the TCP substrate, compared to the hydrophobic PLA uncoated substrate (Figures 6A,B). In addition, Y201 metabolic activity was higher for the PLA-CS-Cat sample compared to the PLA, confirming the fact that this hydrophilic environment is very suitable for cells (Figure 6C).

In addition to the bio-adhesive properties, CS-Cat biomaterial can assume cohesive properties under basic conditions (pH > 8). Upon the addition of the NaIO_4_, the solution became gel in less than a minute. The same cohesive behavior has been exhibited by other types of hydrogels, i.e., polyethylene glycol (PEG)-based gels (Kaneko et al., 2010), in which the deprotonation of the hydroxyl groups in Cat produces the quinone, resulting in cohesive crosslinking reactions (Liu et al., 2006; Hong et al., 2014). Opposite to other reported PEG- Cat hydrogels where multi-arm branched PEGs were primarily utilized to make the sol–gel transition, the formation of hydrogels by using linear polymeric chain arrangement is extremely more challenging, because physical cross-linking of polymer chains can be achieved with several environmental triggers, i.e., temperature, pH and ionic strength, and physicochemical interactions, i.e., hydrophobic, hydrogen bonding, or supramolecular chemistry (Hoare and Kohane, 2008). The first successful work on using natural polymers with this structure has been reported in Hong et al. (2013) where the authors reported the chemical conjugation of HA-Cat via carbodiimide coupling reaction.

The WU is a significant property of a crosslinked hydrogel, especially if the gel is proposed to be used as a bio-adhesive in an internal environment. If the WU of a hydrogel-derived bio-adhesive is elevated, it may embrace an excessive amount of water content from the surrounding tissues and this may cause severe pressure on the surrounding tissues (Spicer, 2020). In this work, we tested two concentrations [10% (w/v) and 20% (w/v)]. The WU test for the 20% (w/v) formulation proved the CS-Cat hydrogel to have a hydrophilic nature, with the ability to hold water molecules, remaining stable (Figure 7B). Furthermore, in terms of mechanical properties, the compression test demonstrated that both the CS-Cat formulations exhibit a Young’s modulus value < 10 kPa (Figure 7C). In particular, the 20% (w/v) hydrogel evidenced a higher E (∼8 kPa) compared to the 10% (w/v) hydrogel (E ∼5 kPa). Both values could be considered suitable for hydrogels intended for soft tissue engineering application, i.e., musculoskeletal (Park H. et al., 2013; Liu et al., 2020), pancreatic (Montalbano et al., 2018), and vascular (Liu et al., 2015) applications. From the obtained enhanced physicochemical and mechanical results, we decided to select the 20% (w/v) concentration to perform the biological tests; indeed, this formulation showed good cell affinity when cells were encapsulated within it. In fact, it presented good properties in terms of cells viability as demonstrated by the Live/Dead assay (Figure 8A) and nuclei integrity (stained with DAPI; Figure 8C). Regarding the Alamar blue assay (Figure 8B), it has been observed a decrease in cells metabolic activity at day 7 of culture. This behavior could be explained considering that, when onto a 2D surface, MSCs are in a proliferative state, which involves a mixed metabolism (glycolysis and oxidative phosphorylation), while within a 3D environment higher rates of glycolysis and a suppression of oxidative phosphorylation are showed due cells shift to differentiation state (Pattappa et al., 2011). This mechanism is influenced by the presence of low oxygen tension which activates the hypoxia-inducible factor (HIF) mediated pathway (Liu and Ma, 2015). Therefore, this may occur in this change of metabolic state of cells within the CS-Cat hydrogel after 7 days of culture.

## Conclusion

A novel CS-Cat biomaterial has been successfully engineered with bio-adhesiveness and cohesiveness properties at different conditions suitable for regenerative medicine, demonstrating excellent cytocompatibility assessed by Y201 cells. The CS-Cat formulation as bio-adhesive showed to improve considerably the hydrophilicity properties of different substrates, with a potential use in diverse conditions during clinical operations, such as for coating orthopedic implants. Furthermore, due to its cohesive properties at high pH and its ability to undergo a sol/gel transition in less than 60 s with an intrinsic porous structure, the CS-Cat formulation has also prospects for being used as a bioink for future bioprinting applications for tissue regeneration (i.e., cartilage tissue), alone or as a hybrid bioformulation in combination with fillers to obtain increased mechanical properties.

## Data Availability Statement

Data supporting this publication are openly available under an “Open Data Commons Open Database License.” Additional metadata are available at: http://dx.doi.org/10.25405/data.ncl.12102666. Please contact Newcastle Research Data Service at rdm@ncl.ac.uk for access instructions.

## Author Contributions

AF and PG conceived the study. AF, ED, and PG designed the experiments. AF, AS, MB, and PG optimized the CS-Cat formulation. ED and SC performed the XPS analysis. FC performed the NMR and UV–Vis analyses. AS performed the mechanical properties. AS and MB performed the *in vitro* cell tests. All the authors analyzed and interpreted the data and prepared the manuscript.

## Conflict of Interest

SC was employed by company Jaber Innovation s.r.l. The remaining authors declare that the research was conducted in the absence of any commercial or financial relationships that could be construed as a potential conflict of interest.
