# Supplementary material for: pH-Triggered Adhesiveness and Cohesiveness of Chondroitin Sulfate-Catechol Biopolymer for Biomedical Applications
Source: Front Bioeng Biotechnol. 2020 Jun 29;8:712. doi: 10.3389/fbioe.2020.00712 (PMC7336602; doi:10.3389/fbioe.2020.00712)
Supplement: Supplementary file 1 [file Data_Sheet_1.pdf]

Supplementary Material

Morphological Analysis by Scanning Electron Microscopy

Morphological analysis was performed on the surface of different substrates after CS-Cat spin-coating by Scanning electron microscopy (SEM, HITACHI TM3030, Maidenhead, UK). The surface of all the substrate appears smooth with the CS-Cat homogenously distributed. This is due to the process parameters condition of the spin-coating technology that allows to create very thin and flat coatings.

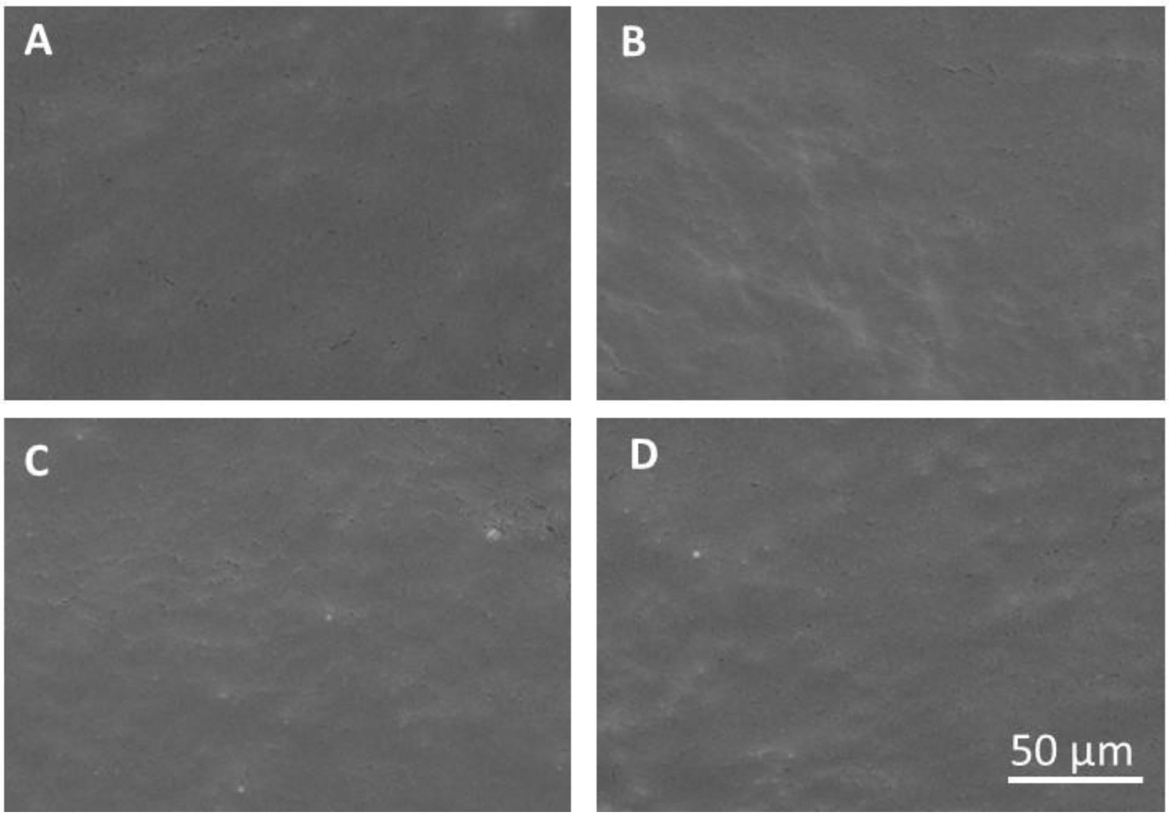

Supplementary Figure 1. SEM images of the surface of the different substrates after CS-Cat coating: (**A**) glass, (**B**)TiO_2, (**C**) PCL and (**D**) PLA.

***In vitro* degradation tests**

The degradation degree of samples (cylindrical shape with dimensions of 0.6 cm diameter and 0.4 cm height) was calculated gravimetrically using an analytical balance (Sartorius, Sartorius AG). Particularly, freeze dried samples (lyophilisation took place for 48 h in Christ ALPHA 1-2/LD Plus, Martin Christ, Germany) were initially weighted and then immersed into 3 mL of PBS (Sigma Aldrich, UK) at pH 7.2 at 37°C. The solution was refreshed three times per week up to 28 days. Following each time points, samples were removed from the buffer solution and freeze dried for re-weighting sample mass. The degradation degree (DD) was calculated by using the following formula:

$$DD\% = 100 \times (W_t - W_0)/W_0$$

where W_0 is the initial dry weight of the hydrogel and W_t is the dry weight at specific time. Values are expressed as the average of three replicates.

The *in vitro* degradation tests showed that the 10% w/v sample displayed a fast increase of the degradation degree with a complete dissolution after 14 days of incubation in PBS, while the 20% w/v evidenced a superior stability with a higher degradation rate after 20 days with almost fully dissolution in 4 weeks.

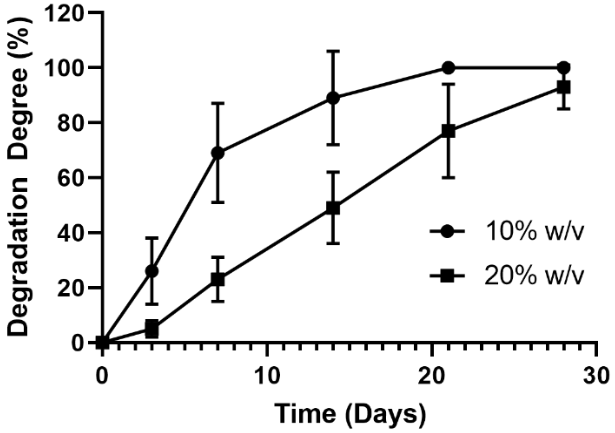

Supplementary Figure 2. Dissolution degree (DD) of the 10% and 20% w/v hydrogels after incubation in PBS at 37°C.
